# Deployment of ACT antimalarials for treatment of malaria: challenges and opportunities

**DOI:** 10.1186/1475-2875-7-S1-S7

**Published:** 2008-12-11

**Authors:** Christopher JM Whitty, Clare Chandler, Evelyn Ansah, Toby Leslie, Sarah G Staedke

**Affiliations:** 1 Department of Infectious and Tropical Diseases, London School of Hygiene & Tropical Medicine, Keppel St, London WC1E 7HT, UK; 2 Joint Malaria Programme, KCMC, Moshi, Tanzania; 3 Dangme West District Health Directorate, Ghana Health Service, P.O. Box DD1, Dodowa, Ghana; 4 HealthNet-TPO, Kabul, Afghanistan; 5 MU-UCSF Research Collaboration/Uganda Malaria Surveillance Project, Mulago Hospital, PO Box 7475, Kampala, Uganda

## Abstract

Following a long period when the effectiveness of existing mono-therapies for antimalarials was steadily declining with no clear alternative, most malaria-endemic countries in Africa and Asia have adopted artemisinin combination therapy (ACT) as antimalarial drug policy. Several ACT drugs exist and others are in the pipeline. If properly targeted, they have the potential to reduce mortality from malaria substantially. The major challenge now is to get the drugs to the right people. Current evidence suggests that most of those who need the drugs do not get them. Simultaneously, a high proportion of those who are given antimalarials do not in fact have malaria. Financial and other barriers mean that, in many settings, the majority of those with malaria, particularly the poorest, do not access formal healthcare, so the provision of free antimalarials via this route has only limited impact. The higher cost of ACT creates a market for fake drugs. Addressing these problems is now a priority. This review outlines current evidence, possible solutions and research priorities.

## Background

The corner-stone of case-management of malaria is the early identification and treatment of those with mild disease with an effective antimalarial. In principle, almost all deaths due to malaria in children and pregnant mothers are avoidable, since patients can be treated with antimalarial drugs which are now available in every country. For a long time, clinicians were restricted to monotherapies which, whilst highly effective in the 1960s, had gradually lost their efficacy due to drug resistance, particularly in Asia and subsequently in East Africa. A prolonged debate about how quickly to introduce artemisinin combination therapy (ACT) largely revolved around the issues of cost and sustainability; their efficacy was never in doubt [1-3]. The advent of the Global Fund and substantial funding for purchase of ACT means that most countries in Africa and Asia feel able to change over to the use of ACT as a policy.

Translating policy into practice and ensuring that ACT reaches the majority of children and vulnerable adults with malaria has, however, proved very challenging. This is not particularly surprising. Prior to the introduction of ACT, it was already known that many, and the majority in some settings, of children with clinical malaria did not receive an antimalarial of any sort. Substituting ACT for existing first-line antimalarials, even if completely effective, was therefore likely to have limited impact, although limited impact is far preferable to no impact at all. For the impact of ACT (and any successors to ACT) to be closer to their potential, policymakers will have to think seriously about how best to improve delivery and targeting of antimalarials. If this is achieved, it could lead to lasting health gains. However, it is important to note that in some settings the epidemiology of malaria is changing, generally for the better [4]. This is entirely welcome and the introduction of ACT may have played a significant part. It does, however, mean that it cannot safely be assumed that some of the strategies, which worked when malaria was by far the most common cause of potentially fatal febrile illness, will necessarily remain appropriate. This review will outline the evidence for the problems with getting antimalarials to the right people, examine some of the potential solutions that have been suggested and finish by considering some of the issues which may emerge as malaria incidence decreases either due to attempts to eliminate or substantially control malaria or for other reasons.

### Evidence for the mis-targeting of antimalarials

There is overwhelming evidence that the majority of those with malaria, particularly in Africa, do not get antimalarials effectively. This applies to all antimalarial classes, not just to ACT, and is, therefore, a major systems failure. Evidence from across Africa demonstrates that there are multiple steps at which delivery of antimalarials may fail. Evidence from Asia and South America is sparser, but lessons from Asia may be important as the burden of disease in some parts of Africa reduces. The first step is that many children and adults with febrile illness that could be malaria are treated at home, often with ineffective treatment [5-7]. Of those who are taken for some form of assessment, the majority are taken to the informal sector [8]. What constitutes the informal sector varies for each local setting and can include traditional healers, chemical or medicine sellers or small shops that sell drugs or other unregulated providers. Of those who do present to the informal sector, the proportion receiving an antimalarial varies. In most informal settings there are no diagnostic facilities available and targeting is largely random: in some, less than 20% of those with symptoms compatible with malaria are given an antimalarial [9]; elsewhere, it can be as high as 75%, although ineffective treatment with monotherapy is common [10] and prescriptions may be in sub-therapeutic doses, prescribed by unqualified staff [11,12]. Of the minority who do access formal health care, malaria is the default diagnosis and an antimalarial will usually be prescribed [13], although this will not necessarily be an effective antimalarial: non-ACT antimalarials continue to be prescribed in the public sector in areas where the local alternative monotherapy has convincingly failed [14]. Once ACT use becomes the norm the proportion treated with ineffective drugs tends to decline [15,16]. The reasons for this are complex [17], and simple solutions are unlikely to be effective. The continuing use of failed drugs is probably even more common in the private sector where price is a major issue, and the continuing use of artemisinin monotherapy is a serious worry for the development of drug resistance [18].

There are multiple barriers to accessing formal healthcare and unfortunately these are likely to fall most on the poorest, who, in the majority of cases, are those most likely to have malaria [19]. These barriers include perceived quality of care [20], lack of knowledge [21,22], distance from health services [23], transport costs, treatment costs [22,24], difficulties in finding care for other children if the parent or guardian accesses care, opportunity costs (which may make up the majority of formal healthcare episode costs to the family) and, sometimes, difficulties for mothers in getting permission and funds to attend health centres [25-28]. Even where drugs are not subsidized, the indirect costs of care exceed the direct costs [29]; free drugs do not solve this problem. Local conceptualization of disease also affects the decision to use formal healthcare [30-32].

### Most of those who are given an antimalarial do not have malaria

There is now good evidence from across Africa and increasingly from Asia that, in most formal healthcare settings where ACT are provided, a substantial proportion, and often the majority of antimalarials are given to children and adults who have no parasites and, therefore, do not have malaria [33,34]. This is also likely to be true in the informal sector. In low-transmission settings, as little as 1% of those given an antimalarial actually have parasites and even in high transmission settings the majority of those given an antimalarial do not have parasites [35]. This has been found to be more of a problem in adults than children although data are sparse. The problem of over-diagnosis is, therefore, likely to increase as malaria incidence declines. It is not just a problem for malaria; significant bacterial causes of fever are likely to be missed [36]. Where microscopy is available it usually only has a limited impact on treatment decisions [33,34]. Several studies demonstrate that those with a positive slide are almost inevitably given an antimalarial and those with negative slides are also in half or more of cases given an antimalarial, which raises interesting questions as to why the slide was requested in the first place. Providing rapid diagnostic tests (RDT) for malaria has been shown to have limited impact [33,35], although in some situations, where prolonged training was provided or significant supervision is available, it can trigger a change in prescribing practice. The reasons for over-diagnosis of malaria are multifaceted, and any attempts to change practice will have to take this into account [37]. Designing strategies to address this is not made easier by the fact that national and WHO guidance on this subject remain ambiguous, essentially supporting a policy of testing for malaria in young children, but providing antimalarial treatment regardless of the test result if the clinician suspects malaria. Since clinicians almost always have these suspicions, the outcome is inevitable.

### The problem of fake drugs

Sub-standard drugs, including antimalarials, are widespread in Africa and Asia. Completely fake drugs are rare where drugs are cheap. ACTs have a much higher retail value than older monotherapies, so the difference in the cost of manufacturing a fake drug and purchase cost provides potential for considerable profits. Fake artesunate is now widespread in South-East Asia, with studies in some countries reporting the majority of artemisinin drugs being fakes [38]. Counterfeit drugs have already penetrated the African market to some degree at least [39]. The fakes are highly sophisticated, including convincing packaging, holograms and marketing, and represent a major technical and law-enforcement challenge [40]. Every malaria patient treated with fake ACT is in danger of progressing to severe illness and in some cases of dying. On the wider scale, if fake artemisinins penetrate the market to a great degree, they may precipitate a collapse of confidence in ACT. Currently, the only thing that prevents this becoming a major public health tragedy in Africa is the relatively high cost of fake ACT, which make it unaffordable to the poorest, who are the group with the highest risk of true malaria. Increasingly sophisticated methods of detecting fake drugs are a major step in the right direction, but will only be effective when linked to a functioning drug-control system and legal sanctions that have a realistic chance of being applied.

### Costs, price and the private market

The majority of the poorest people cannot afford to access healthcare in the formal sector. This is due to transport and opportunity costs in particular [29]. The time of year when malaria is most common in most parts of Africa (during the rainy season) is also the period when the opportunity costs are highest and the transport is most difficult. Only in a minority of settings in Africa is formal healthcare the major provider for acute febrile illness in children. [41]. In reality, the private informal system (meaning shops rather than private formal doctors) provides the bulk of care in many countries. There is evidence that shopkeepers, if properly trained, can improve their targeting of antimalarials [42] and this intervention can be made cost-effective [43], but in the private market ACT drugs remain very expensive, with prices of over 10 USD per treatment not being uncommon. This provides a double-jeopardy, with the poorest not being able to afford to access the formal health care sector, where drugs are free, or heavily subsidized, and not being able to afford antimalarials which are effective in the private sector even if they are properly diagnosed, which they often are not. Serious attempts are now being made to design a subsidy (the Affordable Medicines Fund malaria, (AMFm) which could be effective in reducing the price of antimalarials in the private sector in the periphery to address this problem, but this is a challenging undertaking. ACT is cost effective in almost all settings where there is drug-resistance, which in practice means almost everywhere provided they are targeted to the right people [29]. This is primarily due to the fact that future expensive episodes of malaria, or in the worst case hospital admissions, are avoided by intervening early. Cost-effectiveness does not necessarily translate into willingness to pay for them at or near their market price [44]. Those who have children with febrile illness are often not willing, or able, to pay the current retail price of ACT available in the private sector.

### Potential solutions and gaps in current knowledge

A number of solutions exist to each of the problems identified above. Policymakers currently do not know which are the most effective or, more realistically, which ones are most effective in what settings. The local healthcare structure, epidemiology of malaria and socio-economic factors are all likely to play a major part in determining what is appropriate in a given setting. Unfortunately, far more effort has gone into determining which antimalarials are efficacious than determining how to deliver them effectively, and health services are now having to play catch-up. A drug which is 90% efficacious but delivered to only 20% of those who need it clearly has limited effectiveness at an operational level.

For access, the solutions fall broadly into three categories. The first is to promote the use of home- or community-based care. The principle of this is for early recognition and treatment of malaria by volunteers or individuals with limited training and support, equipped with stocks of antimalarial drugs, which they can prescribe. In such programmes, febrile children are typically treated presumptively with antimalarials. The proximity to the home in comparison to formal healthcare settings can reduce barriers to people accessing formal healthcare. Recognizing that many antimalarial treatments are distributed through the private sector, which is often used in preference to formal services even when these are easy to access [7], the second solution is to build upon this practice and improve the services of the private sector. The third solution is to improve the existing formal healthcare systems, so that people are more likely to use them, and to reduce or remove all out-of-pocket payments to reduce the direct cost of care. All three of these broad approaches have advocates and in practice they are not mutually exclusive and their relative effectiveness will depend on the setting.

Home-based care in rural environments has been demonstrated to have an impact on morbidity [45] and mortality [46], but several other studies failed to find an impact or found limited impact. The evidence around this has been recently reviewed in *Malaria Journal *[47]. No data on the health impact of home treatment with ACT are yet available, although evidence from pilot studies suggests that the delivery of ACT through this route is operationally both feasible and acceptable [48,49]. The disadvantages of delivering ACT through the home-based care route are that it will inevitably increase wastage of the drug and is likely to drive forward resistance. Set against this, it certainly would increase the number of people that get an ACT, although at this stage it is not clear whether that would be an increase in people who get them appropriately. It is likely that home-based care is most useful in areas where malaria is one of the most common causes of acute febrile illness and in rural areas where physical access to formal healthcare is problematic. It is not clear how sustainable this is as a system for delivering ACT on a wide scale.

Improving the service of the informal private sector (meaning shops rather than meaning private doctors) may require better regulation of drug sellers and shops alongside training [12]. Trials of training interventions have had promising results for improving prescribing behaviour of drug sellers in Kenya [42]. The biggest barrier to using ACT widely in the private sector is their very high cost in that sector. The possibility of a global subsidy has been raised [2,50] which, in addition to the current subsidy for public sector distribution from the Global Fund, would encompass all ACT distribution channels, thus minimizing their cost and (it is hoped) bringing them down to the price of any other antimalarial. There are a number of major technical challenges, not least of which is to find sufficient donor enthusiasm to ensure sustainability, but if ACTs could be provided cheaply through the private sector, the possibility of distributing ACTs through shops in hard-to-reach areas would become realistic. Key to this would be maintaining the low cost of ACTs throughout the distribution chain and there needs to be careful investigation of various methods of doing this at every stage.

Where the quality of service in the public sector improves, and this is perceived by the local population, there is evidence of increasing access and demand [51,52]. Incorporating patient education into consultations has been suggested to improve recognition of malaria symptoms, compliance with drug regimens and potentially earlier access to formal sector care. Providing treatment free-of-charge also leads to a change in healthcare utilization [53], although challenges with informal payments for healthcare remain. These may be overcome if health workers are provided with additional financial support from their employer. What is currently lacking is evidence that this translates into improved malaria-associated health outcomes.

All three of these approaches need to be tried both operationally and in comparison with one-another in settings with different transmission intensities and healthcare systems. There is the potential to waste a lot of drugs and resources for very little health benefit if they are not underpinned by adequate research before deployment.

### Improving targeting

In settings where microscopy is already available, there is now clear evidence that simply deploying rapid diagnostic tests or other technologies makes relatively limited difference. Where training and supervision can be provided, prescriber compliance with the results of tests can be improved, but it is likely that far more complex measures are likely to be needed to change prescriber behaviour sustainably. Introducing rapid diagnostic tests can be highly cost-effective [54], when the results lead to a change in prescribing practice [55]. The reasons that, in the formal sector, the prescription of antimalarials is almost a default for any child and, in reality, any adult for whom there is no alternative diagnosis, even in the absence of parasites on microscopy or rapid diagnostic tests, are complex. Further investigation into the reasons for this behaviour is likely to be needed before it is possible to design complex interventions to tackle it. This may involve training of healthcare workers, novel approaches to providing incentives and changing the perceptions of patients. Challenges to training schemes for improving the targeting of ACT by formal sector health workers include the complexity of current guidelines [56] and the perception that patients want antimalarials even when there is no evidence that this is true [57].

### Fake drugs

There needs to be an investment in developing rapid testing for fake drugs at point-of-purchase or nearby. This needs to be linked to epidemiological sampling, which maximizes the chances of identifying fake drugs early on. Relatively little is known about the flow of drugs from areas where fakes are manufactured through to potential target markets in Africa and understanding this better will be key to early identification of the problem.

### Asia

Whilst the majority of effort tends to be concentrated in Africa, because that is where the burden of falciparum malaria is greatest, Asia presents specific challenges for deployment of ACT. Many of the problems found in Africa of low coverage, poor targeting and monotherapy in the private sector are replicated in Asia [58]. In South-East Asia, there is already a concern about an increasing tolerance to artemisinins by parasites [59]. Putting artemisinins in combinations can slow, but is unlikely to stop the development and spread of this tolerance and, if it becomes significant, the implications would be very severe. South-East Asia is also the current focus of fake drugs. On the other hand, the very successful use of diagnostic services, both microscopy and RDTs, in South-East Asia may provide models for other settings. In South Asia, the major problem is two-fold; the majority of malaria is vivax malaria and the minority of febrile illness is malaria, even in areas considered highly endemic. ACT drugs do work against vivax [60,61], but this is generally an expensive and inefficient approach to treating a disease, which can be very well treated in most cases with chloroquine. The problem is that diagnostic services are not widely accessible and, where they are available, species differentiation into falciparum and vivax malaria is not always reliable, particularly in those areas where malaria is most common. Policymakers are therefore left with two options: either improving diagnosis and species differentiation so that ACT is targeted to those with falciparum malaria, or a more blanket approach, which will lead to substantial wastage of the drugs, either on people who do not have malaria, or who have vivax malaria and could be treated with much cheaper alternatives for which there is no likely global shortage. Since falciparum is the minority species and malaria a minority cause of potentially serious febrile illness, the blanket approach is also likely to have a limited public health effect, or at worse a detrimental effect on treatment of febrile illness, much of which is bacterial. This is not a trivial problem: since there are more people living in potentially malaria-endemic areas of India, Pakistan, Bangladesh and Afghanistan than there are in Africa, even relatively small wastage of drugs could have substantial impact on supply.

Wide-scale deployment of RDTs in Asia is one possibility, but the current technology for species-specific RDTs is not well developed for field use, is expensive, and lacks data to demonstrate the advantages (economic and clinical) of this strategy over existing services or alternatives (i.e. microscopy). Where most febrile illness is not malaria, deployment of RDTs will also result in negative diagnosis in the majority of cases. Detection of falciparum will also be rare, since this is the minority species. At the operational level, this may result in similar problems to those found in Africa, where antimalarials are prescribed regardless, as clinicians come to disbelieve what are, in fact generally correct negative results. In most areas of Asia, transmission of disease is unstable and varies by season and geographical area [62]. One solution may be to take advantage of this instability by deploying the cheaper and more robust falciparum-specific RDTs and ACT at community level at the beginning of the falciparum transmission season in areas of endemicity. To deploy this strategy effectively in underserved areas at community level will require the generation of effectiveness data in the operational setting and efficient logistics systems.

### Elimination and the reduction of malaria-impact on deployment of ACT

Malaria incidence appears to be decreasing in a number of countries in Africa [63,64]. Deployment of ACT is likely to have played a part in this, especially in moving from low to very low transmission, due to the gametocidal activity of the drugs. This wholly welcome news does, however, make some of the problems highlighted above more, rather than less, challenging. Home-management of malaria (HMM, HBMF) and other methods which increase the syndromic management of febrile episodes with ACT is likely to have diminishing returns as some countries move to a situation where only a minority, and eventually only a small minority of those with potentially serious causes of fever have malaria. Reduction in prevalence of malaria at presentation will exacerbate the problem of poor targeting in the formal healthcare sector. In time, it may be possible to alter the perceptions of doctors about the risk of malaria. This will have to be handled carefully, or endemic countries could move from a situation where most of those given ACT do not have malaria but all true cases are treated, to one where cases of true malaria are missed. The reduction in the incidence of malaria is likely additionally to have an impact over time on immunity, and the age-range where missed cases could prove fatal may increase. At present, in much of Africa, it is possible to target most activities (eg HMM) to children under five years of age and pregnant women. This may not remain sensible policy in the longer term, which significantly increases the logistical challenges and potential costs.

## Conclusion

The ACT drugs have the potential to transform the current state of malaria in Africa and elsewhere. Simply deploying them using current channels will, however, lead to a limited impact. It is essential that as much effort is put into investigating new ways of delivering drugs to those who need them, as has gone into developing the drugs in the first place. This needs to be followed by a rational policy discourse based on the assumption that complex problems are likely to need complex solutions. There is always a tendency to look for quick fixes based on a single solution, but there is no evidence that this will work in the case of ACT deployment. The policy, therefore, needs to be turned into practice in a sustainable way, and funding secured for this. This is no small task but the potential benefits are great and it has to be undertaken.

## Competing interests

The authors declare that they have no competing interests.

## Authors' contributions

This review arose from discussions between all the authors. CW wrote the first draft, and all authors contributed equally to subsequent drafts. All authors read and approved the final manuscript.
